# Muscular Dystrophies at Different Ages: Metabolic and Endocrine Alterations

**DOI:** 10.1155/2012/485376

**Published:** 2012-06-03

**Authors:** Oriana del Rocío Cruz Guzmán, Ana Laura Chávez García, Maricela Rodríguez-Cruz

**Affiliations:** Laboratorio de Biología Molecular, Unidad de Investigación Médica en Nutrición, Hospital de Pediatría, Centro Médico Nacional Siglo XXI, IMSS, 06703 Ciudad México, DF, Mexico

## Abstract

Common metabolic and endocrine alterations exist across a wide range of muscular dystrophies. Skeletal muscle plays an important role in glucose metabolism and is a major participant in different signaling pathways. Therefore, its damage may lead to different metabolic disruptions. Two of the most important metabolic alterations in muscular dystrophies may be insulin resistance and obesity. However, only insulin resistance has been demonstrated in myotonic dystrophy. In addition, endocrine disturbances such as hypogonadism, low levels of testosterone, and growth hormone have been reported. This eventually will result in consequences such as growth failure and delayed puberty in the case of childhood dystrophies. Other consequences may be reduced male fertility, reduced spermatogenesis, and oligospermia, both in childhood as well as in adult muscular dystrophies. These facts all suggest that there is a need for better comprehension of metabolic and endocrine implications for muscular dystrophies with the purpose of developing improved clinical treatments and/or improvements in the quality of life of patients with dystrophy. Therefore, the aim of this paper is to describe the current knowledge about of metabolic and endocrine alterations in diverse types of dystrophinopathies, which will be divided into two groups: childhood and adult dystrophies which have different age of onset.

## 1. Introduction

There are about 30 different types of muscular dystrophies caused by alterations in diverse genes, which are characterized by the progressive loss of muscle in accordance with age of onset, severity, and the group of muscles affected [1]. The altered protein in most of these dystrophies is located in muscle fiber and is linked to other proteins, enzymes, or extracellular matrix [2]. Myopathologies are associated with different ages of onset, for example, Duchenne muscular dystrophy (DMD) and Becker muscular dystrophy (BMD) as well as Emery-Dreifuss muscular dystrophy (EDMD) demonstrating their first clinical manifestations during childhood [3], whereas some laminopathies such as myotonic dystrophy or limb-girdle muscular dystrophy are developed during adulthood [4]. This group of diseases can cause different physical symptoms such as contractures and scoliosis, respiratory impairment, swallowing and feeding difficulties, and, in some cases, metabolic alterations have been reported [4, 5]. Even so, the primordial clinical sign is muscle weakness [4, 5].

Skeletal muscle is responsible for 70–80% of whole body insulin-stimulated glucose uptake, disposal, and storage; therefore, this tissue is considered the major “player” in energy balance [6]. Furthermore, skeletal muscle has influence on the metabolism and storage of lipids and plays an important role in hormone signaling in insulin sensitivity [6]. Insulin receptors are localized in the cell membrane of target tissues (liver, adipose tissue, and muscle fibers) [7, 8]. It is important to emphasize that in dystrophinopathies the cell membrane of myocytes is damaged [3]. We have hypothesized that dysfunction of membrane of myocytes could alter the insulin receptor function, and as a result an increase may occur in the risk for developing insulin resistance. This fact will be discussed later. Insulin resistance is an important factor for the development of type II diabetes and a risk factor for cardiovascular disease, dyslipidemia, hypertension, and obesity [6, 9]. The progression of muscular damage depends on protein mutation [3]. However, it is important to consider that muscle damage may be age related. The aim of this paper is to describe the current knowledge about of metabolic and endocrine alterations in diverse types of dystrophinopathies, which will be divided into two groups: childhood and adult dystrophies which have different age of onset.

## 2. Childhood Dystrophies

### 2.1. Clinical Characteristics of Duchenne/Becker Muscular Dystrophy

DMD has an incidence of 1/3500 live male births [10]. Initial symptoms begin in early infancy, presenting with muscular weakness between 2 and 4 years of age [11]. This appears when the patient begins to walk [12]. These hip weaknesses will be manifested in an awkward walk with a tendency to fall; later, stair climbing becomes difficult [12]. A classic sign of muscular weakness is Gower's sign, which indicates weakness of the pelvic girdle muscles [11, 12]. Usually, joint contractures at the ankles and hips are increased as the disease progresses [11, 12]. Finally, the development of muscle atrophy leads to wheelchair dependency, which typically occurs between 9 and 12 years of age [10]. The susceptibility for lung infections increases and respiratory capacity decreases [13]. Respiratory insufficiency appears at ~14 years of age and may result in death at ~25–30 years of age [13]. Another cause of death may be cardiomyopathy, which begins at ~5 years of age and evolves in a parallel manner with progression of the disease until this condition eventually leads to the patient's death [14]. Even so, cardiomyopathy is responsible for ~60–80% of deaths [14].

Eagle et al. [15] reported a mean age of death of 14.4 years in the US during the decade of the 1960s, and this value increased to 25.3 years of age in 1990. Fortunately, advances in the treatment of dystrophinopathies have improved life expectancy because patients may reach adulthood in the third and in some cases the fourth decade of life [15].

Becker muscular dystrophy (BMD) is clinically similar to DMD but is a less severe form of myopathy, affecting 1/30,000 males [16]. Patients with BMD start to show clinical signs between 2 and 20 years of age. Compared to DMD, progression in BMD is slower [17, 18]. Some BMD patients present clinical signs similar to those of DMD, whereas some patients are still able to walk at the age of 60 years [19]. Inability to walk prevails at about 30 years old, and death is frequently present 30 years after the appearance of the first clinical signs [20]. Cardiomyopathy usually occurs in 73% of BMD patients >40 years old [20].

Symptoms of Emery-Dreifuss muscular dystrophy (EDMD) generally appear during the first decade of life [21]. The principal clinical characteristics are contractures of the neck extensor muscle, spine, Achilles tendons, and elbows [22]. After muscle weakness, wasting appears during the end of the second decade of life and begins in a humeroperoneal distribution [23]. Cardiac complications begin at the end of the second decade, and sudden cardiac death due to ventricular dysrhythmia is common in this dystrophy [24].

**Figure 1:**
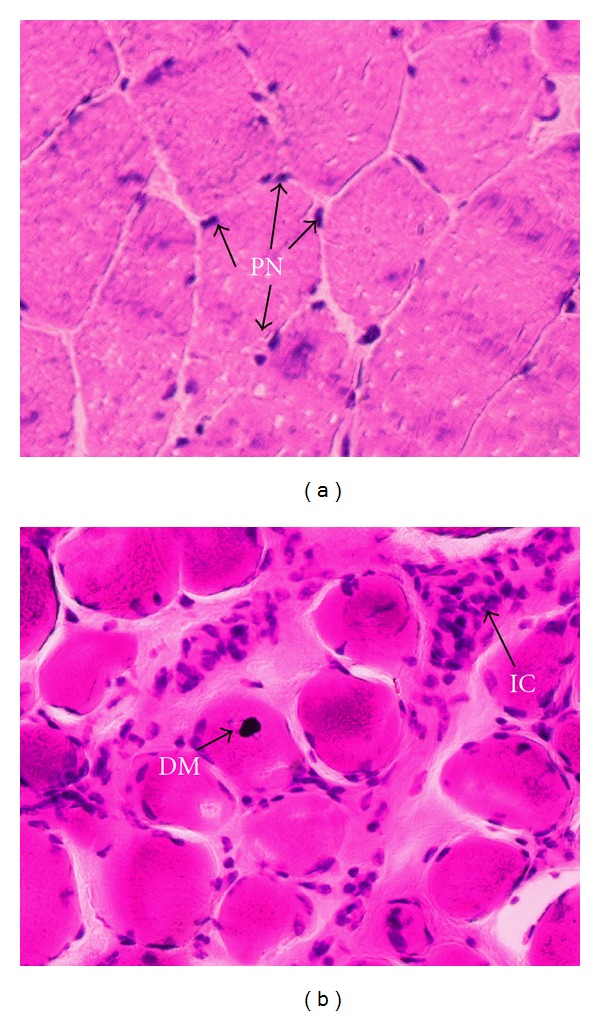
Histology of necrotic skeletal muscle. Image (a) showed a normal skeletal muscle from quadriceps muscle, characterized by healthy myofibres with peripheral nuclei (PN). Skeletal muscle of control subjects was obtained from males without dystrophinopathies at 40 years old. Image (b) showed a necrotic skeletal muscle from quadriceps muscle of a patient with DMD/DMB at five years old, characterized by infiltrating inflammatory cells (ICs) and degenerating myofibres (DMs). Transverse muscle sections stained with haematoxylin and eosin. Scale bar represents 50 m. This biopsy was obtained for the purpose of performing diagnostic.

DMD and BMD are X-linked diseases caused by mutations in the DMD gene, which is responsible for encoding dystrophin protein and is located at locus Xp21 [25–27]. Dystrophin protein is associated with an oligomeric protein complex known as dystrophin-glycoprotein complex or dystrophin-associated protein complex [28, 29]. The mechanical function of the dystrophin-glycoprotein complex is to stabilize the plasma membrane (sarcolemma) during the stress of repeated contraction and relaxation cycles [30]. In patients with DMD, dystrophin is absent in the sarcolemma, whereas in BMD its expression is greatly reduced but is still located in the sarcolemma [31]. Mutations (deletions, duplications, point mutations) cause the lack or semilack of dystrophin [32]. These mutations have many functional and structural consequences in skeletal muscle, in DMD patients; muscle biopsy characteristically demonstrates necrotic or degenerating muscle fibers [32]. These necrotic fibers are surrounded by macrophages. Small immature centrally nucleated fibers are also observed, reflecting muscle regeneration from myoblasts [31–33] that results in a balance between necrotic and regenerative processes in the early phase of the disease. Later, the regenerative capacity of the muscles appears to be exhausted, and muscle fibers are gradually replaced by connective and adipose tissue (Figure 1) [33].

EDMD is usually inherited as an X-linked recessive disorder, although an autosomal dominant form has also been described [34]. This dystrophy is caused by mutations in the gene for emerin (EMD gene) localized on chromosome Xq28 [35]. Emerin is an integral serine-rich protein of the nuclear envelope inner membrane, which is ubiquitously expressed in most tissues and contains 254 amino acids [36]. The autosomal form of EDMD is caused by mutations in the LMNA gene localized at chromosome 1q21.2-q21.3, which encodes an A-type nuclear lamin [37]. This lamin is an intermediate filament protein associated with the inner nuclear membrane [38]. Emerin interacts with both lamin A and lamin C in the nucleus. It has been proposed that these mutations may result in increased nuclear fragility during mechanical stress or increased susceptibility to apoptosis [39].

### 2.2. Endocrine System

The most important endocrine alteration in DMD/BMD patients is hypogonadism, which has been related to dystrophies [40]. Consequences of this state are delayed puberty, growth failure, osteoporosis, and metabolic abnormalities [40–42]. In 2008, Al-Harbi et al. [40] measured total and free serum testosterone levels in 59 men with different dystrophinopathies. Results obtained showed that 54% had low total testosterone, 39% had low total and free values, and 8% had low free with normal total levels [40]. In addition to Becker and Duchenne dystrophies (*n* = 12), other dystrophinopathies were included such as monotonic dystrophy (*n* = 12), facioscapulohumeral dystrophy (*n* = 11), metabolic myopathy (*n* = 7), and body myositis (*n* = 17) [40]. Interestingly, there were no significant differences in the prevalence of hypogonadism among the various forms of myopathy, even after considering age as a confounder [40]. It has been established that testosterone levels decline with aging, and this is associated with decreased muscle mass and strength in healthy subjects [43]. Testosterone treatment increases muscle mass and strength in older hypogonadal males [44]. The importance of testosterone in the maintenance of muscle mass is critical, and testosterone therapy should be considered when hypogonadism is present [43, 44].

Growth hormone (hGH) has anabolic effects in normal skeletal muscle [45]. It has been suggested in only one study that this hormone plays a role in the pathogenesis of DMD, but there is insufficient evidence to support that idea [45, 46].For instance, in one study, treatment with hGH was administered to DMD patients, but no effect was shown on clinical status and natural history of DMD, either beneficial or detrimental [47]. In addition, Merlini et al. [45] demonstrated in DMD patients with impaired hGH secretion that no association exists between diminished secretion of hGH and different forms of the disease. Interestingly, there is a case report of hGH treatment of a young male with DMD with hGH deficiency who showed improved growth velocity and motor function [45]. Controversies still exist about the benefit and the role of hGH and the resolution in regard to treatment administration with hGH for counteracting short stature. This treatment should be individualized with the objective to obtain improved results [40].

No effective treatment has yet been demonstrated to ameliorate the various consequences of muscular dystrophies [48]. However, in recent years a range of approaches have been developed to correct the genetic defect, restore functional expression of dystrophin, slow disease progression, and improve the quality of life for DMD patients [49]. Those treatments can be categorized into three classes: genetic, cell-based, and pharmacological approaches such as corticosteroids [50].

The use of pharmacological treatment with corticosteroids is justified by the fact that DMD is characterized by aggressive inflammation, and there is strong evidence that this contributes to myofiber necrosis [48, 51]. Until a cure for DMD is found, treatment will involve administration of corticosteroids combined with interventions to alleviate cardiac and respiratory problems [51].

**Figure 2:**
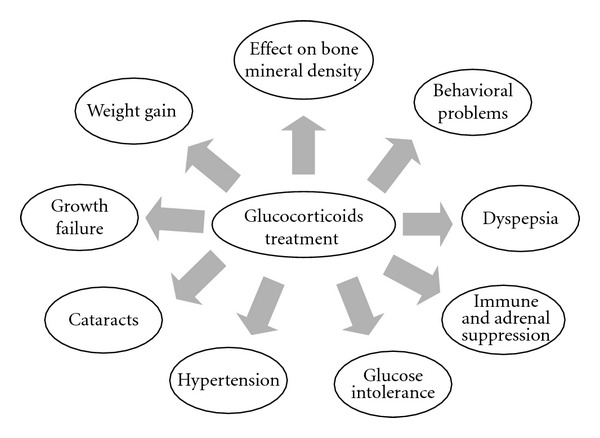
Side effects of glucocorticoid treatment in muscular dystrophies.

However, it is important to note that corticosteroids have a catabolic effect on muscle (nonexercised muscle) and have an effect on preserving existing muscle fibers and reducing inflammation, even though their exact mechanism of action in dystrophic skeletal muscle is unknown [12, 48]. Moreover, use of glucocorticoids [GC] in patients with DMD and BMD is common, but GCs have different side effects (Figure 2) [48]. Two of the most important side effects are growth failure and delayed puberty [52]. Currently, there are no studies examining the outcomes of treating delayed puberty in patients with DMD.

### 2.3. Metabolic Alterations

About a decade ago, clinical researchers began to notice that patients with DMD tended to present obesity [53]. However, it was necessary to demonstrate the prevalence of obesity in children with DMD because obesity had only been described from clinical experience [54]. For this reason, it was also necessary to measure body composition of these patients which, in turn, depended on technology available [55].

 With the advance in knowledge, it was observed that DMD patients also develop malnutrition as a consequence of the progression of the illness [56]. In the following paragraphs, some metabolic issues will be described such as malnutrition and obesity and, additionally, some abnormalities in glucose metabolic pathway.

#### 2.3.1. Malnutrition

In patients with dystrophinopathies, nutrition is a problem to consider. It has been reported that 50% of DMD patients are underweight by the age of 18 [54]. Some studies have shown that this problem generally is caused by feeding difficulties, gastrointestinal dysfunction, and reduced weight gain [57]. It has also been observed that chewing and swallowing difficulties in both ambulant and nonambulant DMD patients are related to increased weakness of masticatory muscles, malocclusion, and other abnormalities of the oropharyngeal process [58]. Additionally, gastric distension has been reported in DMD and BMD, which can contribute to delay gastric emptying, gastroesophageal reflux, and subsequent nutritional disturbances [59].

Malnutrition has also been observed in patients with DMD/BMD who are severely compromised by respiratory failure [13]. This fact has been described in advanced stages of those dystrophinopathies when breathing effort increases and, as a consequence, caloric requirements drastically increase. Gonzalez-Bermejo et al. [13] showed that specific nutritional measures should be taken when patients have advanced forms of dystrophy, and mechanical ventilation becomes necessary because, surprisingly, these patients have balanced energy intakes and resting energy expenditure (REE); hence, they are not likely to suffer from significant malnutrition. However, Gonzalez-Bermejo et al. [13] found that 34% of patients aged  25 ± 4  years who had received nocturnal mechanical ventilation had a decrease of REE. This effect was also observed in patients with DMD of different age groups (10-11 years; 12–14 years; 15–17 years; 18–29 years), where REE was significantly lower than the value obtained for healthy controls [13]. Both the low REE and the low physical activity during the early teenage years result in a low energy requirement and may be related to obesity that frequently occurs in this age group [13]. In contrast, in later stages of the disease, patients increase their basal physical activity [60]. It is possibly due to the presence of respiratory failure, may lead to a high energy requirement, and thus becomes one of the risk factors for development of malnutrition [60]. Additionally, it is important to consider that loss of the ability to self-feed in DMD/BMD patients is very common [61]. The length of meal time also increases with age, ranging from a mean of 18 min in younger patients to a mean of 32 min in older patients [62]. The increase in time is probably related to a combination of increasing weakness of the masticatory muscles and difficulties in chewing and swallowing [62]. Perhaps it would be a good choice to introduce dietary modifications as cutting food into smaller pieces and changing the texture to soft foods in order to facilitate chewing and to reduce meal time [62].

#### 2.3.2. Obesity

As previously mentioned, obesity is an important identified problem in patients with dystrophinopathies [53]. Body mass index (BMI) is the most frequently used indicator in clinical practice in order to make the diagnosis of overweight or obesity [63]. Nonetheless, the main limitation of the BMI is that it does not discriminate between fat mass and lean mass [64]. It has been observed that individuals with a BMI within normal limits have fat mass measurements that fall within values considered as obesity when these have been measured with more precise methods [64]. Therefore, it is necessary to measure fat mass through body composition. It is possible to evaluate the general nutritional status by body weight composition, which could be estimated by measures such as skinfold thickness (ST), dual energy X-ray absorptiometry (DXA), bioelectrical impedance analysis (BIA), and magnetic resonance imaging (MRI) [65].

At the age of 7 years, obesity may occur in patients with DMD, and by the age of 13 years prevalence is 54%, and distribution of body fat is centralized [54]. Moreover, Martigne et al. [56] provided additional information about nutritional status in DMD patients during different ages. This group of investigators studied the progression of nutritional status in 17 DMD patients born prior to 1992. Obesity was evaluated using Griffiths & Edwards charts. According to it, obesity was defined by body weight/age ratio ≥151%. At the age of 13 years, those patients showed a prevalence of 73% for obesity and 4% for underweight. At age 15–26 years, the prevalence of obesity decreased to 47% and prevalence of underweight increased to 34%. Also, obesity at the age of 13 years was associated with later obesity, whereas normal weight status and underweight in 13-year-old patients were predicted later [56].

It has been observed that obesity contributes to the progression of the disease by exerting extra force on already weakened muscle groups, essentially decreasing mobility [56]. Obesity also has implications on increased respiratory involvement as well as poorer psychosocial development [57]. There is one hypothesis that describes the positive association of loss of ambulation and obesity increment in DMD patients [12, 13]. Although Martigne et al. [56] found no relation between the age of ambulation loss and development of obesity.

Generally, fat mass is higher in patients with dystrophy than in healthy subjects [66]. Indeed, as the dystrophic process advances with age, percentage of fat mass increases at least during the first two decades of life [67]. In fact, body composition of DMD patients has been evaluated (by bioelectrical impedance analysis and skinfold-thickness measurement), and the results show that lean mass in DMD (65.3%) was lower (between ~12% and 25%) than in healthy children, whereas fat mass in DMD (31.9% versus 22%) was higher than in control children [67]. Additionally, a positive correlation was found between age and percentage of fat mass and a negative correlation between age and percentage of lean mass [67]. The authors suggest that muscle may have been replaced by connective tissue [67]. In this context, Zanardi et al. [55] studied obese and nonobese children with DMD and evaluated the alteration of body composition in boys with DMD using magnetic resonance imaging. Only obese children showed a markedly increased fat mass (>50%), whereas nonobese children with DMD showed fat mass values similar to healthy boys [55]. In both obese and nonobese children with DMD, the characteristic that was dramatically reduced was muscle mass, showing a reduction of 27% of muscle volume compared with control subjects [55].

It is important to emphasize that the advance in the knowledge and development of new technologies for assessing nutritional status has shown that patients with DMD present abnormal nutritional status such as obesity or malnutrition as part of the natural course of DMD. These alterations are related to advancing age because dystrophy progresses over the course of time.

#### 2.3.3. Glucose and Insulin Metabolism

**Figure 3:**
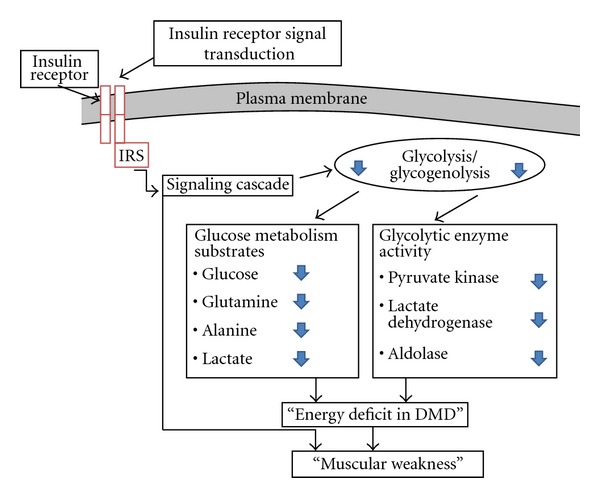
Some aspects of impaired glucose metabolism in patients with childhood muscular dystrophies. The damage in the glucose metabolism, no matter the reason, may probably be one of the causes for energy deficit in DMD patients, and this low energy could result in muscular weakness in muscular dystrophies.

To date, relatively little is known about metabolic alterations in patients with DMD and BMD. However, there are studies that described some disturbances in glucose metabolism, which include reduced glycolytic substrates, reduced activity of glycolytic enzymes, and defects in insulin receptor signal transduction (Figure 3) [68].

Some studies have suggested that there are metabolic differences in the skeletal muscle of patients with DMD and healthy subjects [69]. For example, it has been proven that the concentration of glycolytic substrate glucose, gluconeogenic amino acids such as glutamine and alanine and lactate, a glycolytic product, is lower in DMD patients as compared to controls in skeletal muscle [70]. Also, the decrease in the concentration of lactate in the muscle of DMD patients may be due to the reduction in anaerobic glycolytic activity or lower substrate concentration [71]. As a result, the low concentration of glucose metabolism substrates may be one of the reasons for energy deficit in DMD patients [72].

Nishio et al. also showed that glucose serum concentration in DMD patients is significantly lower and is associated with low creatine kinase activity. This fact may probably be one of the causes of energy deficit in DMD patients [72]. These findings also have a relationship with other causes described in different studies in which glycolytic enzymes were found to have reduced activity in lactate dehydrogenase, aldolase, and pyruvate kinase from muscle biopsies of DMD patients, supporting reduced anaerobic glycolytic activity [68]. Those results are in agreement with the low concentration of previously described glucose metabolism substrates.

Although there are no reports describing whether DMD patients present insulin receptor signal transduction alterations, some studies have evaluated whether there is a defect in insulin secretion or receptor [73]. These studies hypothesized that the possible changes in the sarcolemma of skeletal muscle cells could produce defects in insulin signaling [73]. This has been proposed because patients with diabetes present skeletal muscle weakness that could lead to changes in the sarcolemma as a result of defects in insulin receptor internalization and processing that have been well described in insulin resistance and diabetes [74]. Further research is needed to clarify this hypothesis.

There are other types of evidence that have demonstrated damage of the plasma membrane of myocytes of boys with DMD and BMD [75]. It is well known that membrane properties depend on phospholipid composition [76]. A significant reduction has been observed in muscle biopsies of DMD patients in some membrane components or phospholipids such as total creatine, glycerophosphorylcholine, phosphoryl choline, carnitine, choline, and acetate [68], and lower levels of choline-containing compounds indicate membrane abnormalities. Additionally, a lower ratio of trimethyl amides (TMAs) compared with healthy tissue has been demonstrated in biopsies of DMD patients. TMAs are constituents of phospholipid metabolism and cell membranes, and decreased TMA is considered to be associated with a lower number of cells and reduced rate of membrane synthesis [77]. Decrease in TMA may reflect degenerative changes in the muscles of patients with DMD, resulting in alterations in signal transduction that take place in the sarcolemma [77].

However, despite the above-mentioned evidence, scarce information exists about the alterations in glucose and insulin metabolism in DMD patients. There is only one study by Freidenberg and Olefsky in which an oral glucose tolerance test and the measurement of insulin binding on erythrocytes were performed in DMD patients and age-matched healthy males. The results of this study showed that insulin binding in erythrocytes was 20–30% lower in DMD patients than in healthy subjects. This difference indicated a lower affinity of insulin to its receptor in erythrocytes in DMD patients. This alteration may be present prior to the development of insulin resistance, which may occur in severely immobilized patients. One possible cause for this fact is the increased progression of the dystrophy. Furthermore, in the same study, it was found that patients with DMD had elevated levels of glucose and insulin in comparison to a healthy control group [73].

In summary, in muscular dystrophies which have childhood age of onset, such as DMD and BMD, there are common and well known clinical characteristics and complications that lead the patient to death (Table 1). But also some common metabolic and endocrine alterations have been identified in both DMD and BMD. One of the most important metabolic aspects is nutritional status. Obesity and malnutrition have been identified in these dystrophinopathies. Furthermore, there are some biochemical aspects that have demonstrated an impaired glucose and insulin metabolism. Moreover, these patients present hypogonadism, which is important to emphasize that hypogonadism consequences are related to the age of onset. For instance, since hypogonadism is present in early infancy, these patients are going to present delayed puberty and growth failure (Table 1). However, since DMD/DMB patients have short life expectancy, secondary consequences of hypogonadism such as reduced fertility and oligospermia are less relevant.

## 3. Adult Muscular Dystrophies

Clinical features in muscular dystrophies also can appear in juvenile or adult age, and these phenotypes differ depending on mutation type [78]. Some muscular dystrophies in juvenile or adult age include distal myopathy, Miyoshi and Nonaka [79], inclusion body myositis (IBM) [80], facioscapulohumeral muscular dystrophy (FSHMD) [81], oculopharyngeal muscular dystrophy (OPMD) [82], distal myopathy [83], myotonic dystrophy (MD, type 1 or type 2) [84], and limb-girdle muscular dystrophy (LGMD 1B) [79]. However, we focused this paper on two last dystrophies.

### 3.1. Clinical Characteristics of Myotonic Dystrophy and LGMD

Myotonic dystrophy (MD) is the most common inherited neuromuscular disease in adults, with a global incidence of 1/8000 individuals [85]. Two types of MD exist: type 1 (MD1) and type 2 (MD2) [85, 86]. MD1 is a chronic, slowly progressive, highly variable inherited multisystemic disease. MD1 results from an unstable (CTG) expansion in 3′ UTR of the MD protein kinase gene (MDPK) at 19q13.3 locus [84]. MD2 is caused by an unstable expansion of a CCTG tetraplet repeat in intron 1 of the ZFN9 gene localized on chromosome 3q21.3 [84]. The phenotypes of MD1 and MD2 have a broad spectrum of clinical signs that include mainly myotonia and muscle weakness. The first neuromuscular symptoms appear during a wide age range (20–60 years) [86]. Other clinical signs of these dystrophies include cataracts prior to 50 years of age, cardiac conduction defects, endocrine changes, testicular atrophy, insulin resistance, and hypogammaglobulinemia [84]. In MD2, clinical features appear in adulthood (median age 48 years) in contrast to adult-onset MD1 and childhood onset [87]. The majority of patients (63%) die between 50 and 65 years of age [86, 87]. Pneumonia and cardiac arrhythmias are the most frequent primary causes of death, each occurring in 30% of patients, which was much higher than expected for the general population [88].

Other types of dystrophy are LGMD, which describes a heterogeneous group of muscle disorders characterized by a predominant proximal distribution of limb-girdle, shoulder, and hip weakness [79]. At least 15 different genetic forms of LGMD are now known [79]. The phenotypes begin during early childhood to late adulthood [89]. The LGMD group is still growing today and consists of 19 autosomal dominant and recessive forms (LGMD1A to LGMD1G and LGMD2A to LGMD2M) [84, 89]. The proteins involved are diverse and include sarcomeric, sarcolemmal, and enzymatic proteins [90].

### 3.2. Endocrine Alterations

**Figure 4:**
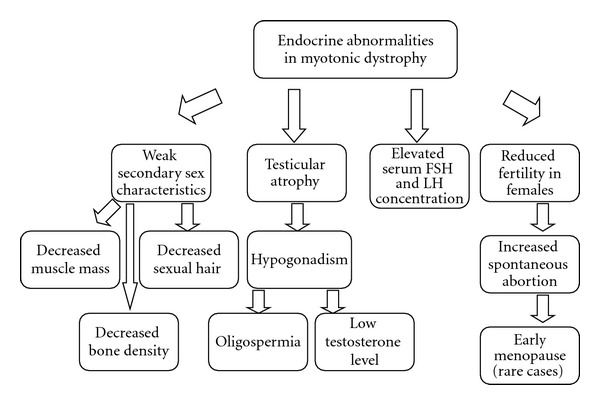
Endocrine abnormalities described in MD1/MD2.

Endocrine abnormities have been reported in MD2 and MD1 patients (Figure 4), including the alteration in testicular function such as hypogonadism leading to oligospermia [91], low levels of testosterone (T), and reduced spermatogenesis [92], resulting in reduced male fertility. In fact, hypogonadism may be a cause for erectile dysfunction (ED), which has been demonstrated in 25% of patients with MD1 [93]. The occurrence of ED is independent of the patient's age but may be related to other intrinsic factors of MD1 such as disease duration and severity and CTG expansion [94, 95]. Also, those patients present elevated levels of follicle-stimulating hormone (FSH) and reduced T level in comparison to control subjects [92, 93, 96].

Hypogonadism is very common in males with myopathies and involves both interstitial (androgenic) and tubular (spermatogenic) gonadal functions. In primary hypogonadism, luteinizing hormone (LH) increases and T level is reduced [93, 94]. In contrast, in compensated hypogonadism, LH increases and T levels are normal [93].

Testicular atrophy is reported to be the most prominent feature in ~80% of MD1 patients [97, 98]. Testicles of MD1 patients are characterized by an increase in the number and size of Leydig cells as well as tubular atrophy, hyalinization, fibrosis of the seminiferous tubules and reduced spermatogenesis. It has been reported that 46% of MD1 patients show hormonal evidence of interstitial gonadal failure [97, 99].

### 3.3. Metabolic Alterations

#### 3.3.1. Malnutrition

Although MD patients demonstrate disorders of the oropharyngeal cavity, myotonia of the tongue and pharynx, impaired pharyngeal contraction, and slowing of esophageal peristalsis, to date, there have been no reports regarding the malnutrition state related to MD [4]. Loss of ability to cut and manipulate food leads to a loss of the ability of a person with MD to ensure adequate nutrition [100]. In this context it has been reported that 62% of patients with MD1 do not meet their daily energy requirements according to government recommendations: 55% of MD1 patients had a fat intake higher than the acceptable macronutrient distribution ranges [4]. Furthermore, 10% of the MD1 group of patients were categorized as obese (BMI > 30) and 13% had BMI values <18.5, which is in the underweight category [4]. Patients with MD1 have macronutrient and energy intake deficiencies as well as an insufficient intake of minerals (copper, zinc, and calcium) [57]. However, there are no reports regarding malnutrition in MD patients.

#### 3.3.2. Obesity

In muscular dystrophy, plasma membrane is damaged, generating myofibers passing through cycles of deterioration and regeneration until the end of its repair capacity [25, 28–30]. This induces the muscle fibers to be susceptible to the development of necrosis and to be replaced by fibrous connective tissue and adipose tissue [30]. Fibrotic tissue is still considered as lean tissue, so the increase in fat mass may be considered as a reflection of fat involution in muscles [30–32]. Myotonic dystrophy is linked to metabolic syndrome including insulin resistance, increased fat mass, and hypertriglyceridemia [58].

Progressive muscle loss associated with fat infiltration will carry a decrease in motor function and an increase in whole body fat mass index and regional fat-free mass index [55, 56]. Therefore, there is a progressive worsening of disease leading to a decrease of the vital capacity as well as total lung capacity and increases in fat mass [56]. MD1 patients present lower regional (legs, arms, and trunk), fat-free mass index (FFMI), and higher fat mass index (FMI) than healthy individuals [101]. In MD1 patients, a correlation has been reported with an increased total fat-free mass index and decreased motor function and with both decreasing vital capacity and total lung capacity [101, 102].

Aitkens et al. [103] observed that patients with neuromuscular disease showed more cases of obesity and were more sedentary than control subjects (37% versus 34%). However, in this study only 11 patients with neuromuscular disease were analyzed, and only four of the patients had myotonic dystrophy [103]. Therefore, this is not the best reference to determine the prevalence of obesity in MD patients [54]. Recently, Kaminsky et al. [102] studied 106 patients with MD1 (46 males and 58 females) within a range of 55 years of age. These authors reported that the prevalence of obesity was 25.6% and hypertriglyceridemia was 47.6% [102]. The increased fat mass in MD1 patients could be considered a reflection of fat involution in muscles and a link with the metabolic disturbances in these patients [102].

#### 3.3.3. Glucose and Insulin Metabolism

Patients with myotonic dystrophy have alterations in glucose metabolism, and it has been reported that these individuals have insulin resistance as an early manifestation [101]. Insulin resistance is the main cause of glucose intolerance in MD1 and, as a consequence, hyperinsulinemia may coexist such as a compensatory mechanism and may later lead to the onset of diabetes mellitus [101, 103].

 However, the prevalence of diabetes mellitus in MD1 patients has not been proven. Muscle wasting and the low physical activity can make worse insulin resistance and lead to deregulation of protein catabolism [104].

Some hypotheses have been described to explain molecular insulin resistance in MD patients. One hypothesis is in regard to insulin receptor and the two existing isoforms: isoform A (IR-A), which lacks exon 11, and isoform B (IR-B), which includes exon 11 [105]. The insulin receptor B (IR-B) predominates in insulin-responsive tissues such as skeletal muscle. Interestingly, patients with MD1/MD2 express predominant insulin receptor isoform type A (IR-A) in skeletal muscle [106]. With a histological evaluation of MD muscle biopsies, it has been shown that the splicing changes in IR precede histological abnormalities [105]. The results showed that alterations in splicing occur prior to development of dystrophic changes, and this abnormal splicing may be a result of altered RNA binding due to the CUG expansion in the DMPK gene [101, 106].

Another metabolic alteration associated with glucose is abnormal insulin secretion in MD patients with normal insulin sensitivity [101]. This suggests damage to the *β*-cell secretory profile. This damage was represented by increased plasma proinsulin concentrations and a remarkably higher- than-normal early secretory response after oral glucose tolerance test in MD1 patients [107, 108]. A possible reason for this abnormal insulin secretion may be related with the protein kinase and the CUG expansion in the DMPK gene [108]. Protein kinase is involved in the modulation of the Ca^+2^ homeostasis in skeletal muscle cells, and Ca^+2^ homeostasis is crucial for *β*-cell secretion events [109]. If the alteration of calcium metabolism of the skeletal muscle also affects the *β*-cell, then the abnormal pattern of insulin secretion may be related to a malfunction of the MD1 protein kinase [109].

In summary, it has been reported that MD patients present obesity with a prevalence of 25.6% and that it is related to muscle atrophy. Obesity may be related to insulin resistance and metabolic disturbances. However, obesity does not explain damage in glucose metabolism because in myotonic individuals this metabolic alteration has been related to CUG expansion in the DMPK gene, which causes splicing changes in IR. This metabolic alteration has also been related to problems with insulin secretion. Therefore, all types of damage in glucose metabolism in MD patients are related to the problem in the gene itself and not with the problems caused by the pathology. In this context, in DMD it has been noted that the metabolic problems are as a result of the pathology, for example, damage in membrane permeability, problems with glycolytic enzymes, lower glucose metabolism substrates, and changes in the sarcolemma.

On the other hand, it is important to denote that in adult muscular dystrophies such as MD, metabolic abnormalities are similar to those identified in childhood muscular dystrophies (Table 1). Additionally, in MD1 and MD2 some endocrine alterations have been identified (Table 1). One of these alterations is hypogonadism, which has also been identified in childhood muscular dystrophies (DMD/BMD). In this case, since onset is during adulthood, the primary and more relevant consequences of hypogonadism are related to reduced fertility, reduced spermatogenesis, low testosterone levels, and erectile disfunction. In contrast to DMD/BMD, these patients had a normal growth and puberty.

## 4. Conclusions

Clinical and genetic characteristics of muscular dystrophies are diverse but all have one common characteristic: muscular atrophy. The muscle is one of the main tissues that regulates lipid and glucose metabolism by hormones such as insulin. It is important to consider that muscular dystrophies are related to weakness, fatigue, decreased mobility, and reduced physical working capacity. In addition to the muscular atrophy in these pathologies, replacement of skeletal muscle for fat and fibrotic tissue produces a reduction of the muscular mass; therefore, there is an imbalance for all these tissue functions. The combination of increased adiposity and sedentary lifestyle increases the risk for the development of metabolic syndrome. Age is an important factor in the muscular dystrophies, which produces the differences in clinical manifestation because age causes a more rapid progression of clinical, metabolic, and hormonal problems. For example, DMD patients often have short stature, whereas MD patients do not have height-related issues. DMD/BMD patients are frequently wheelchair-bound, and MD individuals do not present this limitation. DMD patients have problems with delayed puberty, whereas MD patients may have reproductive capabilities and have a functional sexual life. Metabolic problems may also increase if clinical manifestations begin in early age as in DMD and BMD. In this type of dystrophy, obesity is observed in the first decade, and during the course of time these patients may show malnutrition, whereas MD patients only develop obesity. Knowledge in regard to metabolic, physiological, and molecular alterations in muscular dystrophies will provide tools that will improve the quality of life for these patients.

## Figures and Tables

**Table 1:** Clinical aspects, metabolic and endocrine issues on muscular dystrophies.

		*Age of onset*	*Clinical characteristics*	*Metabolic issues*	*Endocrine issues*	*Clinical complications*	*Life span*
*Childhood Muscular Dystrophy*	*DMD*	Early infancy	Muscle weakness Gower's sign Joint contractures Muscle atrophy	Obesity Insulin resistance Malnutrition	Hypogonadism Delayed puberty Low testosterone level Growth failure	Wheelchair dependency Loss of ability of self-feeding Gastric distension Respiratory insufficiency Cardiomyopathy	25–30 years old
	*BMD*	2–20 years old					Variable: 4th or 5th decade of life

*Adult Muscular Dystrophy*	*MD1 *	20–60 years old	Myotonia Muscle weakness	Obesity Insulin resistance Hyperinsulinemia Hypertriglyceridemia Glucose intolerance	Hypogonadism Oligospermia Low testosterone levels Reduced fertility Erectile dysfunction Testicular atrophy	Hypogammaglobulinemia Pneumonia Cardiac arrhythmias	Variable: 5th or 6th decade of life
	*MD2*	~48 years old					

## Abbreviations

DMD: Duchenne muscular dystrophy
BMD: Becker muscular dystrophy
EDMD: Emery-Dreifuss muscular dystrophy
DYS: Dystrophin C-terminus
hGH: Growth hormone
GC: Glucocorticoids
REE: Resting energy expenditure
BMI: Body mass index
ST: Skinfold thickness
DXA: Dual energy X-ray absorptiometry
BIA: Bioelectrical impedance analysis
MRI: Magnetic resonance imaging
TMA: Trimethyl amides
IBM: Inclusion body myositis
FSHMD: Facioscapulohumeral muscular dystrophy
OPMD: Oculopharyngeal muscular dystrophy
MD: Myotonic dystrophy
MDPK: Protein kinase gene
LH: Luteinizing hormone
FFMI: Fat-free mass index
FMI: Higher fat mass index
IR-A: Isoform A-Insulin receptor
IR-B: Isoform B-Insulin receptor.
